# Diffuse Idiopathic Skeletal Hyperostosis (DISH)-Phagia: A Case Report and Review of Literature of a Rare Disease Manifestation

**DOI:** 10.7759/cureus.47221

**Published:** 2023-10-17

**Authors:** Diogo Soares, Francisco Bernardes, Marta Silva, José Miradouro, Daniel Lopes

**Affiliations:** 1 Orthopaedics and Traumatology, Centro Hospitalar do Tâmega e Sousa, Penafiel, PRT

**Keywords:** spine, osteophyte resection, osteophyte, dysphagia, diffuse idiopathic skeletal hyperostosis

## Abstract

Diffuse idiopathic skeletal hyperostosis (DISH), also called Forestier disease, is a clinical entity characterized by ossification of the anterolateral ligaments of the spine. DISH is more commonly diagnosed in older males, with an estimated prevalence of 42% in patients older than 65 years. As the disease affects predominantly the thoracic spine, dysphagia is a rare presentation of this entity observed in only 0.6-1.0% of the cases.

We present a clinical case of an 84-year-old male with complaints of progressive dysphagia and foreign body sensation within one year of evolution. Computed tomography imaging revealed an anterior C4-C5 osteophyte compressing the posterior hypopharyngeal wall. Flexible endoscopy revealed a deformed and stenotic hypopharynx. The patient underwent surgical treatment with anterior cervical osteophyte resection using the Smith-Robinson approach. The patient showed significant improvement in preoperative symptoms, and no recurrence was detected at six months of follow-up. We also aim to discuss the clinical and radiological characteristics of the disease, as well as the crucial steps for a correct diagnosis and treatment of this rare disease.

## Introduction

Diffuse idiopathic skeletal hyperostosis (DISH) is a systemic disease characterized by increased ossification and osteophyte formation, affecting ligamentous and tendinous insertions of the spine [1]. The anterolateral aspect of the middle and lower thoracic spine is the segment most affected by the pathology, with primary involvement of the anterior longitudinal ligament [2,3]. The cervical spine is affected in 78% of the cases, most frequently between C4 and C7 levels [4]. The exact etiopathogenesis of DISH remains unknown, although some authors proposed an association with older age, male gender, obesity, type 2 diabetes mellitus, metabolic syndrome, and gout [5-7]. DISH is usually asymptomatic, and the diagnosis is commonly established with the Resnick and Niwayama criteria [1,5,8]. When the upper cervical spine is affected by the disease, the direct mechanical compression by osteophyte growth can cause pharyngeal and esophageal spasms, leading to the development of progressive dysphagia. This manifestation was denominated DISH-related dysphagia, and Curtis et al. were the first to propose the terminology “DISH-phagia” [6].

## Case presentation

**Figure 1:**
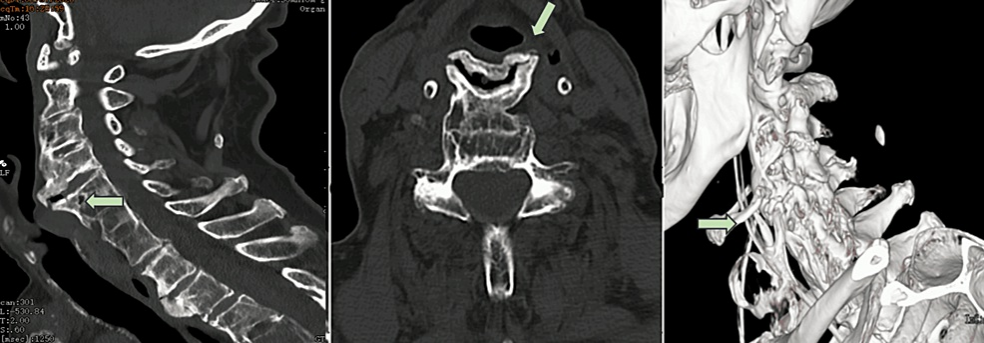
Preoperative CT imaging showing exuberant anterior C4-C5 osteophyte comprising the posterior hypopharyngeal wall. CT, computed tomography

We present a clinical case of an 84-year-old male with a past medical history of hypertension, degenerative aortic valvopathy, and suspicion of initial dementia syndrome referred to neurology consultation. The patient developed complaints of high dysphagia and foreign body sensation within one year of evolution. The dysphagia displayed a progressive pattern, initially restricted to solid foods and deteriorating in the last two months with the development of dysphagia to fluids, food impaction sensation, and significant weight loss (10 kg in two months). There were no other pathological findings on physical examination. The patient was admitted and evaluated by Internal Medicine, Neurology, Gastroenterology, and Otolaryngology. The plain cervical radiographs showed no signs of instability in dynamic views. Cervical computed tomography imaging revealed extensive ossification of the anterior and posterior longitudinal ligaments starting from C3 to C7 along with exuberant anterior C4-C5 osteophyte comprising the posterior hypopharyngeal and high esophageal wall (Figure 1).

**Figure 2:**
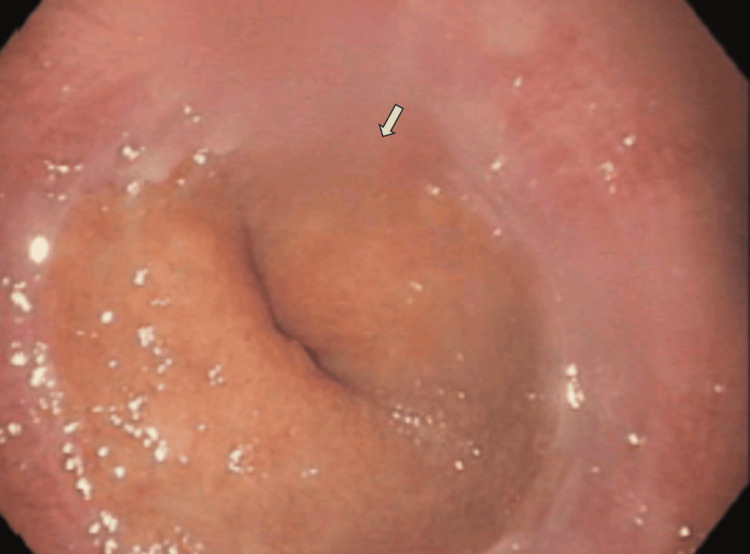
Flexible endoscopy revealing a deformed and stenotic hypopharynx.

Brain MRI revealed no alterations, and central causes for dysphagia were excluded after neurology evaluation. The patient was then submitted to a flexible endoscopy that revealed a deformed and stenotic hypopharynx with no mucosal lesions, suggesting extrinsic compression of the hypopharynx and high esophagus (Figure 2). The classical diagnostic criteria for DISH include three major elements: the presence of vertebral flowing ossifications present at a minimum of four contiguous vertebrae, preservation of disc height, and lack of significant degenerative changes at the involved vertebral segments, which differentiates DISH from degenerative spondylosis. The absence of ankylosis at the facet-joint interface as well as the absence of sacroiliac joint erosion, sclerosis, or fusion differentiate DISH from ankylosing spondylitis.

**Figure 3:**
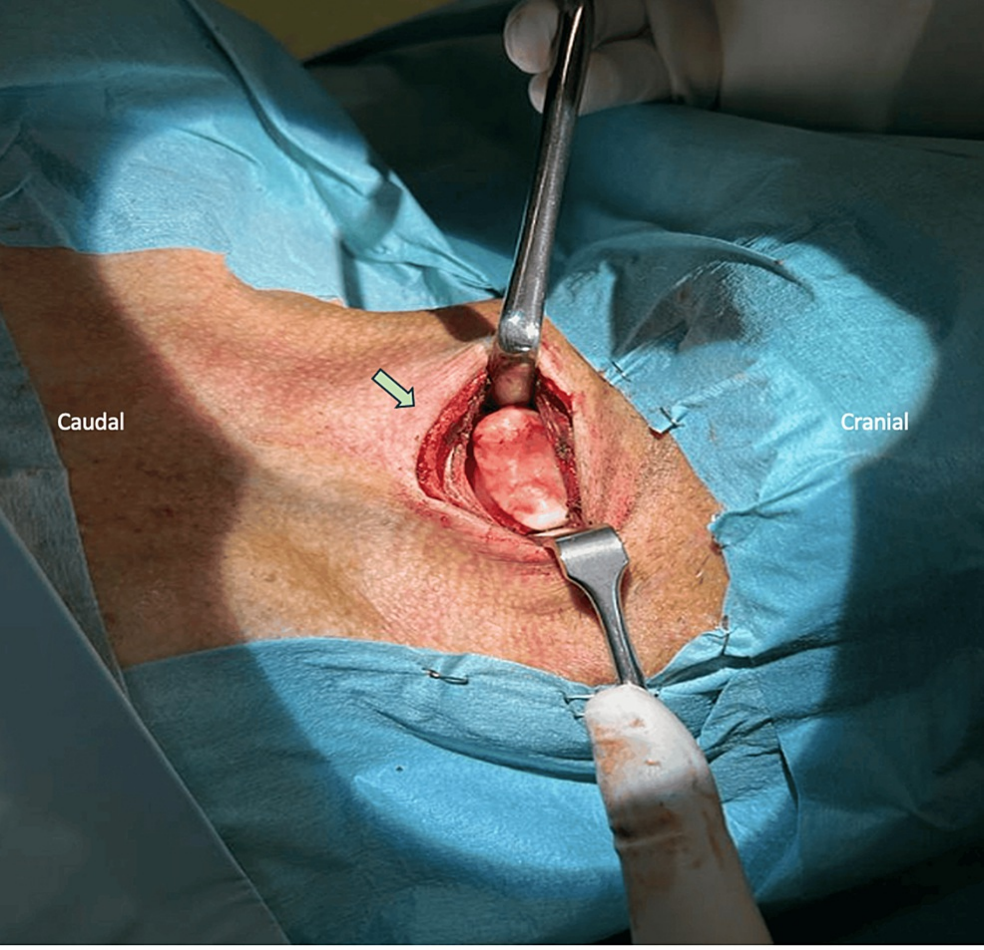
Intra-operative picture showing exuberant anterior C4-C5 osteophyte.

Dynamic swallowing study with barium contrast elicited molding of the posterior wall of the cervical esophagus with delayed contrast progression. Laboratory tests including HLA-B27 were normal. According to the Resnick and Niwayama criteria, the diagnosis of cervical DISH with dysphagia due to extrinsic compression of hypopharyngeal and esophageal was established [1]. Considering the progressive dysphagia leading to severe weight loss, the patient underwent surgical treatment with anterior cervical osteophyte resection between C4 and C5 performed via the Smith-Robinson approach, using Sonopet (Stryker, Kalamazoo, MI) to allow safer bone dissection (Figure 3).

**Figure 4:**
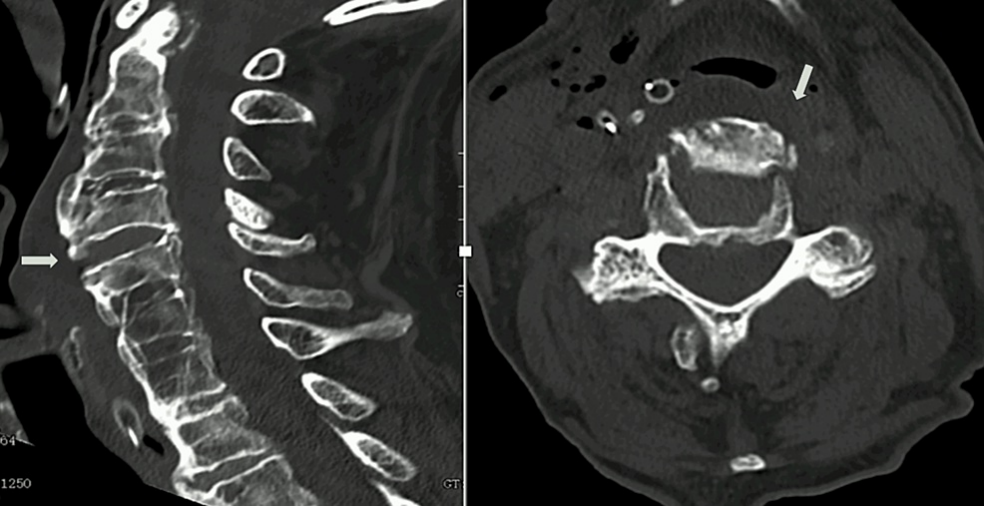
Postoperative cervical CT scan revealing adequate removal of cervical osteophytes responsible for extrinsic esophageal compression.

Due to the advanced age of the patient and the stability of the cervical spine in dynamic radiographs, no fusion or instrumentation was performed. The postoperative period was uneventful, with no acute complications observed. The fluid dysphagia resolved immediately after surgery, meanwhile the difficulty in swallowing solid foods persisted in the early postoperative period. A postoperative cervical CT scan revealed that the cervical osteophytes responsible for extrinsic esophageal compression were correctly excised (Figure 4).

The patient underwent a rehabilitation program with swallowing and voice training with gradual improvement in dysphagia. Dysphagia for solid foods gradually improved with the rehabilitation program and was completely resolved after the fourth month after surgery.

## Discussion

DISH is more commonly reported in males and is generally accepted to be a disease related to older age, with a prevalence of 28% in patients over the age of 80, which is compatible with the age group of our patient. DISH is a very rare cause of dysphagia, observed in only 0.6-1.0% of the cases. However, some studies consider that it may be underdiagnosed in the elderly population [9,10].

The proposed pathophysiological mechanisms for dysphagia in DISH are direct hypopharyngeal or esophageal mechanical compression causing an inflammatory process that generates spasms of the pharyngoesophageal region. Therefore, dysphagia typically develops as a result of esophageal compression in lower cervical segments, most frequently at C4-C5. When the upper cervical spine is involved, the larynx is often compressed, causing laryngeal elevation and limited mobility of the epiglottis, which can also be a cause of dysphagia [9]. The diagnosis of DISH is established using the radiological Resnick and Niwayama criteria, which include the presence of calcification and/or ossification along the anterolateral aspect of at least four contiguous vertebral bodies, integrity of intervertebral disc height, and the absence of ankylosis in the interapophysial joints [1,3]. A progressive high dysphagia can be attributed to DISH in a patient that meets Resnick and Niwayama criteria when other causes of dysphagia have been excluded. Cervical computed tomography imaging is considered the gold standard method for diagnosis as it allows the identification and characterization of the anatomical location of cervical osteophyte compression. Dynamic swallowing study with barium contrast is also recommended, as it is a useful examination in determining the degree of esophageal compression [9].

According to the literature, conservative treatment is considered the first line of treatment in mild and moderate disease composed of diet modification, non-steroid anti-inflammatory and myorelaxant drugs, and anti-reflux treatments [11-13]. Classically, surgical treatment is reserved for severe cases with progressive dysphagia and weight loss that do not respond to the conservative treatment, as in the presented case.

Karaarslan et al. stated that it is important to differentiate hyperostotic osteophytes from osteoarthritic osteophytes since it can dictate the choice of the surgical technique [9]. Surgical resection of hyperostotic osteophytes seems to be sufficient in the surgical treatment of DISH, whereas additional stabilization with instrumentation or fusion might be mandatory after the excision of osteoarthritic osteophytes [9]. Von der Hoeh et al. recommend osteophyte resection and intervertebral fusion with PEEK (polyetheretherketone) cages for augmented stability and to prevent osteophyte regrowth [14]. Shriver et al. stated that instrumentation after osteophyte resection may lead to further compression of the esophagus and should not be recommended [15]. Hines et al. hypothesized that upper esophageal sphincter dysfunction may contribute to dysphagia; therefore, cricopharyngeal myotomy may be considered as an additional procedure in the surgical treatment of DISH-related dysphagia [16].

In our case, considering the patient’s age and stability of the cervical spine, no fusion or instrumentation was performed in addition to the osteophyte resection.

The surgical approach most frequently used is the anterior cervical approach (Smith-Robinson) as it is considered to have fewer risks [4]. Although there were no surgical complications in the presented case, this procedure may be associated with potentially serious complications, such as hematoma formation, Horner's syndrome, recurrent laryngeal nerve paralysis, superior laryngeal nerve paralysis, dysphonia, esophageal injury, and cervical instability [9]. Posterolateral and transpharyngeal approaches are the other two approaches described in the literature. The posterolateral approach carries the risk of injury to the internal carotid artery and sympathetic chain, whereas the most feared complication of the transpharyngeal approach is infection due to contamination by the oropharyngeal flora [9].

Few papers have been published to predict surgical prognosis for DISH. Therefore, recurrence rates reported in the literature are very inconsistent. Miyamoto et al. reported a 65% recurrence rate in long-term follow-up, whereas Urrutia et al. and Lui Jonathan et al. reported no recurrence in their series [17-19]. More recently, a single-institutional database study by Chung et al. with 17 cases of DISH-related dysphagia with a mean follow-up of 29.5 months reported a recurrence rate of 18% [18].

## Conclusions

Dysphagia is a very rare presentation of DISH. Surgical treatment is recommended in the setting of progressive symptoms conditioning severe loss of weight. Osteophytectomy and excision of calcifications of the anterior cervical spine, when performed with the appropriate indications, are considered effective treatments for relieving dysphagia in patients with DISH. Due to the possibility of recurrence, long-term follow-up of these patients should be maintained.
